# Shifting Survival Horizons in Advanced Ovarian Cancer: A Conditional Survival Perspective

**DOI:** 10.3390/curroncol33010017

**Published:** 2025-12-29

**Authors:** Aydan Farzaliyeva, Huseyin Akilli, Ozden Altundag, Esra Kuscu, Nejat Ozgul

**Affiliations:** 1 Department of Medical Oncology, Baskent University Faculty of Medicine, 06490 Ankara, Türkiye; oaltundag@baskent.edu.tr; 2 Department of Obstetric and Gynecology, Baskent University Faculty of Medicine, 06490 Ankara, Türkiye; hakilli@baskent.edu.tr (H.A.); ekuscu@baskent.edu.tr (E.K.); nejatozgul@baskent.edu.tr (N.O.)

**Keywords:** ovarian carcinoma, conditional survival, prognosis, long-term survival, disease progression

## Abstract

Advanced-stage epithelial ovarian cancer (EOC) is defined by biological heterogeneity and poor outcomes, and traditional survival metrics fail to reflect the evolving nature of prognosis as patients survive longer. This study aimed to evaluate conditional survival (CS) in advanced EOC using both overall survival (OS) and progression-free survival (PFS) metrics to provide a dynamic understanding of long-term outcomes. We retrospectively analyzed 808 patients with FIGO stage III–IV EOC who underwent surgery at Baskent University Ankara Hospital between 2004 and 2024. CS estimates were calculated for additional 1- and 5-year intervals among patients who had already survived 6 months, 1, 3, or 5 years after surgery. Median OS and PFS were 4.37 and 1.70 years, respectively. Peritoneal dissemination and platinum resistance were independent predictors of poor survival. Approximately 11% of patients achieved survival beyond ten years. The 1-year CS-OS increased from 87% at 6 months to 95% at 5 years, while the 5-year CS-OS rose from 49% to 66%; corresponding CS-PFS values increased from 89% to 95% and from 44% to 62%. Conditional survival analysis underscores that prognosis in advanced ovarian cancer is not static but continually improves with time survived and sustained disease control. These insights redefine long-term outcomes and provide a modern foundation for individualized patient counseling and survivorship planning.

## Simple Summary

Advanced-stage epithelial ovarian cancer (EOC) is one of the most fatal gynecologic malignancies, characterized by marked biological heterogeneity that makes prognostication and patient counseling particularly challenging. Traditional survival statistics fail to capture the dynamic, time-dependent nature of prognosis in this diverse population. In this two-decade real-world study involving a large cohort, we applied conditional survival (CS) analysis to evaluate how prognosis evolves as patients live longer and remain progression-free. Our findings show that survival expectations become increasingly favorable over time, reflecting the dynamic and time-dependent nature of risk. Recognizing this evolving prognosis can help clinicians tailor follow-up intensity and improve the accuracy of patient counseling. Conditional survival thus provides a more realistic, evidence-based framework for personalized survivorship planning and may help reduce uncertainty and anxiety among long-term survivors of advanced EOC.

## 1. Introduction

Epithelial ovarian cancer (EOC) is the most lethal gynecologic malignancy, and nearly 70–80% of patients present with advanced-stage disease at diagnosis [1]. Despite substantial therapeutic progress—including maximal cytoreductive surgery, platinum-based chemotherapy, and the introduction of maintenance therapies such as PARP inhibitors and bevacizumab—long-term outcomes remain unfavorable, and five-year survival rarely exceeds 40% in stage III–IV disease [2,3]. Yet, a small subset of patients achieve survival far beyond a decade, underscoring the profound biological and clinical heterogeneity that characterizes advanced EOC [4,5]. This heterogeneity results in highly variable survival trajectories, where early mortality and durable long-term remission coexist within the same stage, complicating prognostication and patient counseling.

Traditional survival metrics, however, provide only a static prognosis anchored to the time of diagnosis and do not account for the evolving, time-dependent risk patients experience as they survive longer. In advanced EOC, the probability of progression or death changes over time, reflecting a time-dependent risk rather than a constant hazard. Consequently, patients’ expected clinical outlook also shifts over time, creating what is referred to as a dynamic prognosis. Conditional survival (CS) is the statistical method that quantifies this dynamic prognosis by recalculating survival probabilities at successive time points, thereby translating time-dependent risk into clinically interpretable estimates. By incorporating elapsed survival time into risk estimation, CS offers a more individualized and clinically meaningful representation of prognosis—one that aligns more closely with real-world patient management than static Kaplan–Meier estimates [6,7,8].

Despite the conceptual value of CS, existing studies in ovarian cancer have several important limitations. Most prior analyses have included patients across all disease stages, even though recurrence patterns, biological behavior, and competing risks differ markedly between early- and advanced-stage disease [9,10,11]. In advanced-stage EOC—where recurrence is nearly universal and prognosis is highly dynamic—pooling stages may mask clinically relevant differences and underestimate time-dependent improvements in survival [12]. Moreover, previous CS studies have relied almost exclusively on overall survival–based CS (CS-OS), even though progression-free survival (PFS) often serves as the more sensitive and clinically actionable endpoint in advanced disease [9,11,12,13]. This is particularly relevant in the era of targeted maintenance therapies such as PARP inhibitors, which exert their strongest effects on PFS rather than OS [14]. To our knowledge, only one study by Szamreta et al. has evaluated conditional survival in ovarian cancer using both overall and progression-free endpoints; however, this analysis included patients across all disease stages, limiting its applicability to the advanced stage population [10]. As a result, PFS-based conditional survival (CS-PFS) remains critically understudied, and virtually no large, long-term advanced-stage cohorts have examined how prognosis evolves specifically among patients who remain progression-free over time.

Although biological heterogeneity in ovarian cancer is widely acknowledged, its mechanistic linkage to long-term survival variability has rarely been integrated into prognostic frameworks. A deeper understanding of how tumor-intrinsic characteristics, treatment responsiveness, and host factors modulate risk over time is essential for contextualizing conditional survival patterns—particularly the transition from high early mortality to durable long-term remission.

To address these gaps, the present study focuses exclusively on advanced-stage (FIGO III–IV) EOC and evaluates both CS-OS and CS-PFS, offering a comprehensive assessment of how prognosis evolves as patients survive longer or remain free of progression. Additionally, we quantify long-term survival and identify clinical and treatment-related determinants associated with extended survival and recurrence risk. Through this approach, we aim to provide clinicians with a more accurate, time-adapted, and practically useful framework for counseling patients and planning survivorship care.

## 2. Methods

### 2.1. Study Population

This was a single-center, retrospective observational study that included all patients diagnosed with EOC between January 2004 and December 2024 at Baskent University Ankara Hospital. Patients were identified from the institutional oncology registry, which contains validated data on clinical, pathological, and follow-up parameters.

Patients who did not undergo surgery at the study center or who lacked institutional follow-up data were excluded from the analysis. Subsequently, according to the 2018 International Federation of Gynecology and Obstetrics (FIGO) staging system, patients diagnosed with early-stage disease (stage I–II) were also excluded.

After applying these criteria, only patients with FIGO stage III–IV epithelial ovarian cancer who underwent surgery and had complete clinicopathologic and follow-up data were included in the final cohort.

### 2.2. Ethical Approval

Ethical approval for this retrospective study was obtained from the Institutional Ethics Committee of Başkent University in accordance with national research guidelines (approval number: KA25/391, approved on 30 June 2025). Owing to the non-interventional and anonymized design of the study, the requirement for written informed consent was waived by the Institutional Ethics Committee.

### 2.3. Definitions and Outcomes

Baseline assessments included demographic, clinical, surgical, pathological, and treatment-related variables obtained from the institutional oncology database.

Demographic variables comprised age at diagnosis and menopausal status. Clinical and pathological characteristics included comorbidities, histologic subtype, tumor grade, FIGO stage (2018 classification), lymph-node involvement, and peritoneal dissemination.

Surgical outcomes were evaluated based on the extent of cytoreduction and categorized as optimal (residual disease ≤ 1 cm) or suboptimal (residual disease > 1 cm).

Treatment-related parameters included the type of adjuvant chemotherapy and platinum-sensitivity status.

The primary outcome of this study was conditional survival (CS), defined as the probability of remaining alive for an additional *y* years, given that a patient had already survived (or remained progression-free) for *x* years, and was calculated as follows:

CS-OS = OS(*x* + *y*)/OS(*x*)

CS-PFS = PFS(*x* + *y*)/PFS(*x*)

CS-OS represents conditional survival for patients alive at each time interval (*x* years), while CS-PFS indicates conditional survival for those alive and without progression at the same intervals.

Conditional survival was analyzed separately according to overall survival (CS-OS) and progression-free survival (CS-PFS) to capture both overall and disease-free prognostic perspectives. CS estimates were calculated for additional 1- and 5-year survival intervals among patients who had already survived 6 months, 1, 3, and 5 years after surgery.

The secondary outcomes included OS and PFS, as well as their associations with clinicopathologic, surgical, and treatment-related factors.

OS was defined as the time from the date of surgery to death from any cause, and PFS as the time from surgery to the first documented event of disease progression or death, whichever occurred first. The date of progression was defined as the first documented occurrence of any of the following events: disease recurrence, appearance of new metastatic lesions, death, initiation of second-line treatment, radiologic evidence of progression, or clinical documentation indicating disease advancement.

Because this study spans a 20-year real-world cohort, progression events could not be uniformly captured using a single radiologic standard such as RECIST [15]. Therefore, a composite real-world progression definition was adopted, incorporating radiologic findings, clinical documentation, and treatment-based indicators of disease worsening. This approach reflects how progression is identified in routine oncology practice, is consistent with established real-world evidence methodologies, and enhances sensitivity for detecting clinically meaningful progression while remaining broadly comparable to real-world PFS definitions used in previous studies. Long-term survival was defined as survival of ≥10 years after the date of primary surgery.

Patients who were alive or progression-free at the last follow-up were censored at that date.

### 2.4. Statistical Analysis

Baseline characteristics, including patient and disease variables as well as potential covariates, were described using appropriate summary measures. Continuous variables such as age were reported with the median and range, and categorical variables with counts and percentages.

The Kaplan–Meier method was applied to estimate overall survival (OS) and progression-free survival (PFS) from the time of surgery. Associations between patient and disease characteristics and OS or PFS were evaluated using univariate and multivariable Cox proportional-hazards models. Results were reported as hazard ratios (HRs) with 95% confidence intervals (CIs), and a *p* value < 0.05 was considered statistically significant. The proportional hazards assumption was evaluated for all Cox regression models using Schoenfeld residuals. Both global tests and variable-specific tests demonstrated no significant violations, confirming that the proportional hazards assumption was satisfied for all models.

Both OS and PFS were measured from the date of surgery (index date), with censoring at the date of the last follow-up for patients who were alive or had not experienced disease progression.

Conditional survival rates were derived using the Kaplan–Meier method to estimate the probability of surviving an additional 1 or 5 years, provided that no OS or PFS event had occurred during the preceding 6 months and at 1-, 3-, or 5-year time points following surgery.

### 2.5. Censoring Approach

Censoring was applied uniformly across the 20-year study period using standard right-censoring procedures. Patients without an event were censored at their last documented follow-up, independent of diagnosis year or evolving treatment availability. Because censoring was based solely on follow-up completeness rather than treatment era, the likelihood of period-related bias influencing survival or conditional survival estimates is minimal.

The date of progression was defined as the first documented occurrence of any of the following events: disease recurrence, appearance of new metastatic lesions, death, initiation of second-line treatment, radiologic evidence of progression, or clinical documentation indicating disease worsening.

### 2.6. Missing Data Handling

Missing data were managed using standard right-censoring procedures consistent with survival analysis methodology. Patients with missing progression dates were considered progression-free up to the last known follow-up and were censored at that time in the PFS analysis. For cases in which the cause of death was unavailable, overall survival analyses were conducted using all-cause mortality, and all patients with a documented death date were included in OS models regardless of cause. Because the proportion of missing data was low and missingness was not expected to be associated with the outcomes, multiple imputation was not performed. Instead, right-censoring was applied to preserve each patient’s contribution to the risk set and to avoid introducing model-based assumptions. This approach ensures that conditional survival estimates reflect the observed follow-up structure and the timing of events.

Statistical analyses were conducted using IBM SPSS Statistics, version 26.0 (IBM Corp., Armonk, NY, USA).

## 3. Results

**Figure 1:**
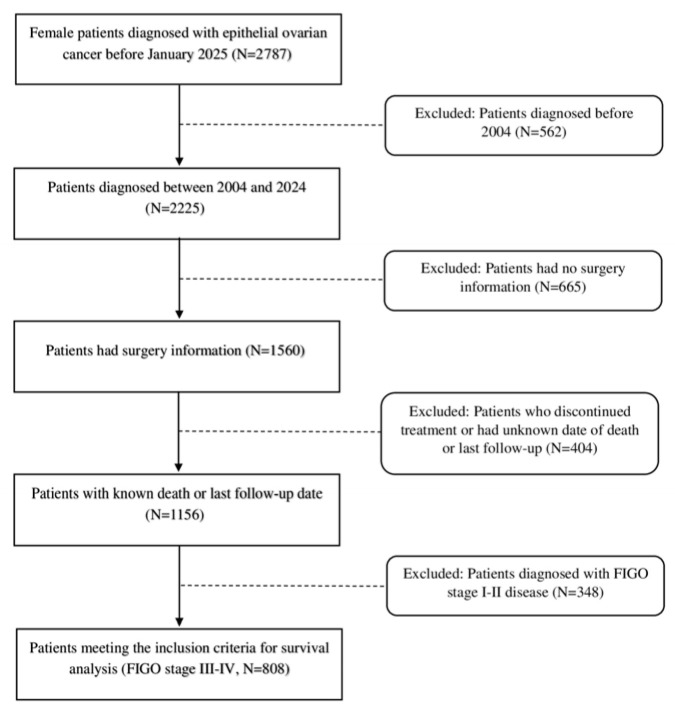
Flowchart illustrating the selection process of patients with advanced epithelial ovarian cancer included in the final analysis from the institutional oncology database.

Between January 2004 and December 2024, a total of 1078 patients with epithelial ovarian cancer who underwent both primary surgery and follow-up at our institution and had complete medical and pathological records were identified. After the exclusion of patients diagnosed with early-stage disease (FIGO stage I–II), 808 patients with advanced-stage epithelial ovarian cancer were included in the final analysis (Figure 1).

Baseline clinicopathologic characteristics of the study population are summarized in Table 1.

The median age at diagnosis was 57 years (range, 23–91), and approximately half of the patients (49.8%) were between 40 and 59 years of age. Most patients (67.1%) were postmenopausal at diagnosis, and 44.9% had at least one comorbidity. A high proportion of tumors in this cohort were of the serous subtype (92.0%), and 83.5% were high-grade serous carcinoma. Similarly, the high frequency of stage III disease (85.5%) and the substantial proportion of patients with peritoneal (81.1%) and lymph-node involvement (67.7%) reflect the typical presentation pattern of advanced EOC. These distributions therefore align with expected real-world characteristics of late-stage disease and support the generalizability of our findings to comparable advanced EOC populations. Regarding surgical outcomes, optimal cytoreduction was more frequent (70.2%) than suboptimal cytoreduction (29.8%). Adjuvant chemotherapy was administered to 81.4% of patients, most commonly with a paclitaxel–carboplatin combination regimen. Details of the applied treatment protocols are provided in Supplementary Table S1. Among evaluable cases, 77.5% were platinum-sensitive, and 22.5% were platinum-resistant.

At the time of analysis, 63.6% of patients had died, and 72.6% had experienced progression. Overall, 67.7% of patients survived less than 5 years, whereas 10.8% achieved long-term survival of 10 years or longer. Baseline characteristics of long-term survivors are summarized in Table 2.

Long-term survivors were generally younger and more frequently premenopausal at diagnosis, and they tended to exhibit more favorable tumor characteristics, including a higher proportion of non-serous and low-grade histology, as well as lower rates of lymph-node metastasis and peritoneal dissemination. From a treatment perspective, they more often achieved optimal cytoreduction, demonstrated greater platinum sensitivity, and experienced fewer recurrences compared with the overall cohort.

The Kaplan–Meier survival estimates showed a median overall survival (OS) of 4.37 years (95% CI, 4.03–4.71) and a median progression-free survival (PFS) of 1.70 years (95% CI, 1.53–1.87).

Overall survival declined sharply within the first few years, dropping from high 1-year estimates to substantially lower 5- and 10-year values, while a similar pattern was observed for PFS (Table 3). These trends indicate that most adverse events occur during an early high-risk window, after which the survival curves flatten, suggesting improved prognosis for patients who remain event-free beyond the initial years.

**Figure 2:**
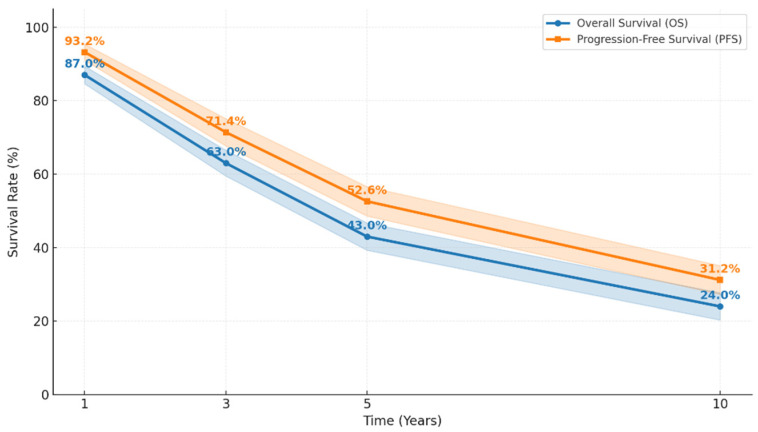
Overall and progression-free survival in patients with advanced epithelial ovarian cancer.

Both OS and PFS curves demonstrated a steep early decline, followed by a relative plateau beyond approximately 5 years (Figure 2). This pattern is quantitatively reflected in the interval-specific survival rates reported in Table 3, which show substantial reductions between years 1 and 5 for both OS and PFS, with a slower rate of decline thereafter.

The results of univariate and multivariate Cox regression analyses evaluating clinical factors associated with PFS and OS are presented in Table 4 and Table 5.

In the multivariate Cox regression analysis for PFS, younger age (<60 years; HR = 0.77, 95% CI, 0.65–0.91; *p* = 0.002), suboptimal cytoreductive surgery (HR = 1.22, 95% CI, 1.03–1.45; *p* = 0.024), peritoneal dissemination (HR = 3.43, 95% CI, 2.56–4.60; *p* < 0.001), and platinum resistance (HR = 4.56, 95% CI, 3.75–5.54; *p* < 0.001) were identified as independent prognostic factors.

In the multivariate model for OS, peritoneal dissemination (HR = 4.14, 95% CI, 2.93–5.85; *p* < 0.001) and platinum resistance (HR = 3.23, 95% CI, 2.63–3.96; *p* < 0.001) remained statistically significant independent predictors of poor survival.

Conditional survival estimates (CS-OS and CS-PFS) are summarized in Table 6.

**Figure 3:**
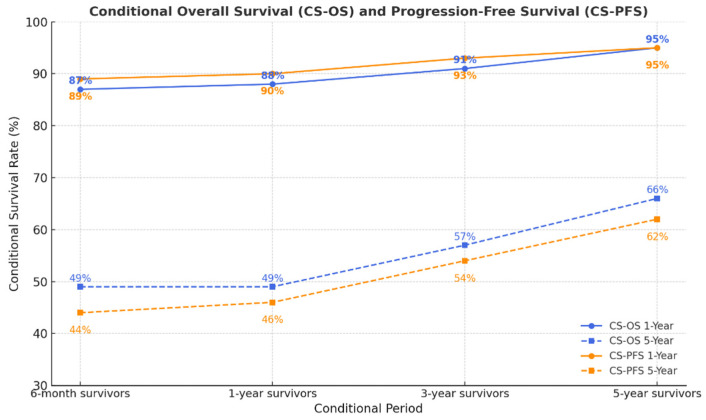
Conditional survival rates based on previous time survived (CS-OS) or on time without progression (CS-PFS) in patients with advanced epithelial ovarian cancer.

Conditional survival estimates showed consistent improvement as patients remained alive or progression-free over time (Table 6). Both CS-OS and CS-PFS demonstrated substantial gains after the first few years, indicating that prognosis becomes markedly more favorable once patients surpass the early high-risk period. This pattern reflects a transition from an initially unstable survival phase to a more stable long-term survivorship state, particularly among women who remain progression-free beyond 3–5 years. These findings demonstrate that, for patients who remain alive and progression-free over time, conditional survival rates substantially improve, particularly beyond the first few years after diagnosis (Figure 3).

**Figure 4:**
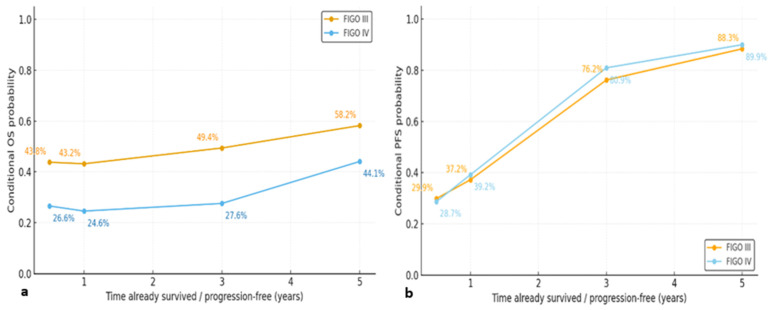
Conditional survival curves stratified by International Federation of Gynecology and Obstetrics (FIGO) stage. (**a**) Conditional overall survival for FIGO stage III and FIGO stage IV disease. (**b**) Conditional progression-free survival for FIGO stage III and FIGO stage IV disease.

We also conducted stage-stratified conditional survival analyses. Stage-stratified conditional survival curves demonstrated that although FIGO IV patients initially exhibited markedly poorer CS-OS (Figure 4a) and CS-PFS (Figure 4b) compared with those with FIGO III disease, these differences progressively diminished over time. As patients remained alive or progression-free for longer periods, the conditional probability of surviving additional years improved substantially in both groups, indicating a convergence of long-term prognostic trajectories. This pattern suggests that early disease burden strongly influences short-term risk, whereas long-term survivors—regardless of initial stage—experience a more stabilized and comparable risk profile.

## 4. Discussion

The present retrospective cohort study provides a comprehensive real-world assessment of conditional survival and time-dependent prognosis in patients with epithelial ovarian cancer (EOC). Our findings demonstrate that both OS and PFS decline steeply within the first few years after diagnosis, reflecting the high-risk early phase of the disease. However, survival tends to stabilize thereafter, and this pattern is visually evident in the survival curves (Figure 2), where both OS and PFS exhibit a clear plateau beyond approximately five years, indicating substantial attenuation of recurrence and mortality risk among long-term survivors. A subset of patients ultimately achieves long-term or even exceptional survival beyond ten years, underscoring marked heterogeneity in tumor biology and treatment responsiveness. Importantly, conditional survival estimates showed a pronounced improvement in the probability of surviving additional years once patients remained alive and progression-free beyond the initial treatment period, highlighting the dynamic and time-dependent nature of prognosis in advanced EOC.

These findings are consistent with contemporary population-based analyses of advanced EOC, which likewise demonstrate a sharp early decline in survival. In our cohort, the most pronounced drop occurred within the first five years after diagnosis, and this pattern persisted across studies despite the incorporation of modern maintenance therapies such as PARP inhibitors and bevacizumab. This suggests that the early high-risk window remains incompletely mitigated despite therapeutic advancements [16,17].Comparisons with external cohorts should, however, be interpreted cautiously, as methodological differences—including the use of surgery date rather than diagnosis date as the index time in our study—may influence absolute survival estimates.

Peritoneal spread was independently associated with poorer outcomes, consistent with previous studies showing that a higher Peritoneal Cancer Index and extensive disease predict non-optimal cytoreduction and reduced survival [18,19]. Likewise, complete or optimal cytoreduction remains a key determinant of prognosis, as residual disease significantly worsens OS [20,21]. Platinum sensitivity also continued to stratify outcomes in our cohort; patients with platinum-resistant relapse had substantially shorter PFS and OS, whereas longer platinum-free intervals were linked to improved survival [3,22]. Overall, these findings align with contemporary evidence, confirming the central prognostic roles of peritoneal involvement, surgical outcome, and platinum response in advanced ovarian cancer.

Long-term survival was achieved in 10.8% of women in our cohort, aligning with recent population-based and single-institution studies reporting similar long-term survival rates in advanced EOC [13,21,23,24]. However, we were surprised to find that the long-term survivor group also included patients with peritoneal dissemination, residual tumor, and platinum resistance. These findings reveal the clinical heterogeneity of long-term survival in advanced EOC and reinforce that such outcomes likely arise from a complex interplay of host immunity, tumor-intrinsic biology, and treatment-related factors rather than any single determinant. This also suggests the presence of specific immunologic and genetic mechanisms associated with exceptional (≥10-year) survival beyond established surgical and clinicopathologic factors [25,26,27,28]. Ongoing multi-omic and immune-profiling efforts aim to delineate these pathways and identify actionable biomarkers. It is noteworthy that individuals who survive longer inherently contribute more follow-up time; consequently, the characterization of long-term survivors in this retrospective cohort may be influenced by survivorship bias and should be interpreted with appropriate caution. In our cohort, CS improved as time accrued, both after remaining alive and after remaining progression-free. The 1-year CS-OS increased from 87% at 6 months to 95% at 5 years, and the 5-year CS-OS rose from 49% to 66%; in parallel, the 1-year CS-PFS rose from 89% to 95%, and the 5-year CS-PFS increased from 44% to 62%. These findings show that as patients remain alive and free of progression over time, their risk of death or recurrence steadily declines. This time-dependent improvement in prognosis cannot be captured by conventional Kaplan–Meier curves calculated from the time of diagnosis, highlighting the clinical value of conditional survival analysis.

Shin et al. (Korean Cancer Registry) reported a 5-year conditional relative survival that improved from 65% to 84% across mixed-stage populations, demonstrating larger absolute gains due to the inclusion of early-stage cases [9]. In contrast, Zheng et al. (SEER, advanced-stage only) observed more modest increases, with 5-year conditional survival rising from 37% after 1 year survived to 44% after 3 years, closely resembling the magnitude seen in our advanced-only cohort [12]. Our findings are also consistent with the recent population-based analysis by Kofoed et al., which evaluated conditional survival in advanced ovarian cancer using Swedish national registry data. In their cohort, the 5-year conditional survival increased from approximately 33% among patients who had survived 1 year after diagnosis to 57% among those who had survived 5 years, mirroring the gradual improvement observed in our advanced-stage population. [13] Complementing these, Kahn et al. reported that the 10-year conditional probability of survival substantially increased among women who had already survived several years, reinforcing the favorable time-dependent shift in prognosis [11].

All these studies evaluated conditional survival solely based on overall survival. To our knowledge, apart from our current analysis, only one study by Szamreta et al. has assessed conditional survival using both overall and progression-free endpoints. In that large U.S. analysis, which computed CS after both time alive and real-world PFS (rwPFS), the adjusted 5-year CS-OS increased from approximately 48% at 6 months to 66% at 5 years, whereas the adjusted 5-year CS conditioned on rwPFS rose more sharply from 54% to 85%, representing a greater absolute gain than in our cohort [10].

The smaller absolute improvement in CS-PFS observed in our study compared with mixed-stage or rwPFS-based cohorts may be partly explained by several factors. First, because our analysis was restricted to advanced-stage disease, patients may have been subject to higher competing risks—such as late recurrence, treatment-related morbidity, or non-cancer-related mortality—which could attenuate conditional gains. However, this interpretation should be viewed cautiously, as we did not perform a formal competing risk analysis. Second, our study used the date of primary surgery rather than treatment initiation as the index time, and progression was defined using stringent clinical and radiologic criteria, which may have produced more conservative PFS estimates. Third, temporal and therapeutic heterogeneity across cohorts—particularly differences in access to maintenance therapies such as PARP inhibitors and bevacizumab—likely contributed to variations in absolute CS values.

Taken together, our results are directionally consistent with contemporary evidence showing that survival expectations improve substantially as patients remain alive and progression-free.

Our stage stratified conditional survival analyses provide novel insights into the dynamic prognostic evolution of advanced stage epithelial ovarian cancer. While conventional survival analyses consistently demonstrate poorer outcomes for FIGO stage IV disease, prior studies have rarely explored how stage specific prognostic differences evolve over time using conditional survival frameworks. In our cohort, the initially pronounced disadvantage observed in FIGO IV patients progressively attenuated among those who remained alive or progression free, resulting in converging long term conditional survival trajectories between FIGO III and IV disease. These findings suggest that although initial disease burden strongly determines early mortality and progression risk, conditional survival captures a clinically relevant shift in risk among long term survivors that is not reflected by traditional baseline survival estimates.

Conditional survival estimates have practical implications for clinical management. The steep decline in OS and PFS during the first several years supports the need for intensive surveillance early after treatment, particularly during the high-risk 0–3-year interval. Conversely, the marked stabilization and plateau in risk after approximately five years suggest that follow-up intensity could reasonably be reduced in long-term survivors. Conditional survival information may also enhance prognostic counseling by allowing clinicians to provide individualized, time-updated expectations—for example, reassuring patients who remain progression-free for several years that their likelihood of future survival has substantially improved. Incorporating CS into routine survivorship discussions may therefore improve decision-making, reduce uncertainty, and support more patient-centered care.

This study has several limitations. It was based on retrospectively collected single-institution data, which may restrict the generalizability of our findings. Because all patients were treated and followed at a single tertiary oncology center, selection bias is possible. Stratification by histologic subtype would be clinically informative; however, in the present cohort, high-grade serous carcinoma accounted for the vast majority of serous epithelial ovarian cancer cases, and serous histology was the dominant subtype overall. As a result, the number of non-high-grade serous and non-serous tumors, including endometrioid, clear cell, mucinous, and other subtypes, was very limited. Consequently, event counts were insufficient to support statistically robust or interpretable Kaplan–Meier or conditional survival analyses, and additional subgroup analyses by histologic subtype were not applicable. This distribution is consistent with the well-established predominance of high-grade serous carcinoma among patients presenting with advanced-stage epithelial ovarian cancer reported in contemporary epidemiologic studies. The study spans a 20-year period during which treatment standards evolved substantially; however, the treatment era was not incorporated into the statistical models, and we were unable to adjust or stratify outcomes by therapeutic period. Therefore, unmeasured era effects may have influenced survival estimates and should be considered when interpreting the findings. Despite these limitations, our study has several notable strengths. It includes a large and well-defined cohort of advanced EOC treated and followed over an extended period within a specialized center, providing reliable insight into long-term outcomes. The consistent surgical indexing and comprehensive institutional records ensured accurate data capture and minimal information loss. Unlike most previous reports, we assessed conditional survival using both overall (CS-OS) and progression-free (CS-PFS) endpoints, offering a more realistic and clinically meaningful view of how prognosis evolves over time. Moreover, unlike studies including all-stage ovarian cancer, our analysis focused exclusively on advanced-stage disease, in which defining prognosis and providing accurate counseling remain particularly challenging.

Collectively, these strengths affirm the robustness of our findings and underscore the value of this work in elucidating the shifting survival horizons of advanced ovarian cancer through a conditional survival perspective.

## 5. Conclusions

Conditional survival analysis shows that prognosis in advanced ovarian cancer improves substantially as patients remain alive and progression-free, offering clinically useful, time-updated insights for counseling and survivorship planning. While these findings provide a meaningful framework for real-world practice, their broader applicability should be interpreted with caution, given the single-center retrospective design, and confirmation in multi-institutional cohorts is warranted.

## Figures and Tables

**Table 1:** Baseline characteristics of patients with advanced epithelial ovarian cancer (*n* = 808).

Characteristics	Group	*n* (%)
Age (years)	<40	65 (8.0)
	40–59	402 (49.8)
	≥60	341 (42.2)
Age (years)	Mean ± SD/Median (Min–Max)	57.05 ± 12.13/57 (23–91)
Menopausal status	Premenopausal	266 (32.9)
	Postmenopausal	542 (67.1)
Comorbidities	No	445 (55.1)
	Yes	363 (44.9)
WHO Histological Subtype	Serous	743 (92.0)
	Endometrioid	25 (3.1)
	Clear cell	18 (2.2)
	Mucinous	12 (1.5)
	Others (undifferentiated, mixed, carcinosarcoma)	10 (1.2)
Tumor grade	Low grade	133 (16.5)
	High grade	675 (83.5)
FIGO stage	III	691 (85.5)
	IV	117 (14.5)
Surgery	Optimal	567 (70.2)
	Suboptimal	241 (29.8)
LN involvement	No	261 (32.3)
	Yes	547 (67.7)
Peritoneal involvement	No	153 (18.9)
	Yes	655 (81.1)
Chemotherapy	No	150 (18.6)
	Yes	658 (81.4)
Platinum sensitivity	Sensitive	595 (77.5)
	Resistant	173 (22.5)
Survival status	Alive	294 (36.4)
	Dead	514 (63.6)
PFS event	No	218 (27.4)
	Yes	578 (72.6)

Abbreviations: FIGO—International Federation of Gynecology and Obstetrics; LN—lymph node; SD—standard deviation; WHO—World Health Organization.

**Table 2:** Characteristics of long-term survivors (≥10 Years) among patients with advanced epithelial ovarian cancer (*n* = 87).

Characteristics	Group	*n* (%)
Age (years)	<40	10 (11.5)
	40–59	49 (56.3)
	≥60	28 (32.2)
Age (years)	Mean ± SD/Median (Min–Max)	53.63 ± 11.32/52 (24–80)
Menopausal status	Premenopausal	38 (43.7)
	Postmenopausal	49 (56.3)
Comorbidities	No	45 (51.7)
	Yes	42 (48.3)
WHO Histological Subtype	Serous	14 (16.1%)
	Endometrioid	28 (32.2%)
	Clear cell	21 (24.1%)
	Mucinous	13 (14.9%)
	Others (undifferentiated, mixed, carcinosarcoma)	11 (12.6%)
Tumor grade	Low grade	25 (28.7)
	High grade	62 (71.3)
FIGO stage	III	77 (88.5)
	IV	10 (11.5)
Surgery	Optimal	62 (71.3)
	Suboptimal	25 (28.7)
LN involvement	No	33 (37.9)
	Yes	54 (62.1)
Peritoneal involvement	No	46 (52.9)
	Yes	41 (47.1)
Adjuvant chemotherapy	No	13 (14.9)
	Yes	74 (85.1)
Platinum sensitivity	Sensitive	82 (97.6)
	Resistant	2 (2.4)
Survival status	Alive	76 (87.4)
	Dead	11 (12.6)
PFS event	No	47 (54.0)
	Yes	40 (46.0)

Abbreviations: FIGO—International Federation of Gynecology and Obstetrics; LN—lymph node; SD—standard deviation; WHO—World Health Organization.

**Table 3:** Overall and progression-free survival rates in patients with advanced epithelial ovarian cancer.

Time Point (Years)	OS (%, 95% CI)	PFS (%, 95% CI)
1	87.0 (84.6–89.4)	93.2 (91.0–95.4)
3	63.0 (59.5–66.5)	71.4 (67.7–75.1)
5	43.0 (39.3–46.7)	52.6 (48.6–56.6)
10	24.0 (20.3–27.7)	31.2 (27.3–35.1)

Abbreviations: OS: overall survival; PFS: progression-free survival.

**Table 4:** Univariate and multivariate Cox regression analyses for progression-free survival (PFS) in patients with advanced ovarian cancer.

Parameters	Univariate		Multivariate	
	*p*	HR (95% CI)	*p*	HR (95% CI)
Age, years (<60/≥60)	**0.032**	0.83 (0.71–0.99)	**0.002**	0.77 (0.65–0.91)
Menopausal status	0.928	0.99 (0.84–1.18)	–	–
Comorbidities (yes/no)	0.101	0.87 (0.74–1.03)	–	–
Histology (serous/non-serous)	**0.024**	1.47 (1.05–2.05)	0.166	0.78 (0.56–1.11)
Tumor grade (low/high)	0.822	0.98 (0.78–1.21)	–	–
FIGO stage (III/IV)	0.257	1.14 (0.91–1.44)	–	–
Surgery (optimal/suboptimal)	**0.000**	1.44 (1.23–1.70)	**0.024**	1.22 (1.03–1.45)
LNM (yes/no)	0.313	1.10 (0.92–1.31)	–	–
Peritoneal involvement (yes/no)	**0.000**	3.98 (3.02–5.26)	**0.000**	3.43 (2.56–4.60)
Platinum sensitivity (sensitive/resistant)	**0.000**	5.61 (4.61–6.81)	**0.000**	4.56 (3.75–5.54)

Bold text indicates statistical significance at *p* < 0.05 level. Abbreviations: LNM—lymph node metastasis; FIGO—International Federation of Gynecology and Obstetrics.

**Table 5:** Univariate and multivariate Cox regression analyses for overall survival (OS) in patients with advanced ovarian cancer.

Parameters	Univariate		Multivariate	
	*p*	HR (95% CI)	*p*	HR (95% CI)
Age, years (<60/≥60)	**0.000**	1.40 (1.18–1.67)	0.158	1.17 (0.94–1.44)
Menopausal status	**0.000**	1.40 (1.16–1.68)	0.182	1.17 (0.93–1.47)
Comorbidities (yes/no)	0.075	1.17 (0.98–1.39)	–	–
Histology (serous/non-serous)	0.146	1.28 (0.92–1.79)	–	–
Tumor grade (low/high)	0.192	1.17 (0.93–1.48)	–	–
FIGO stage (III/IV)	**0.000**	1.72 (1.38–2.14)	0.280	1.15 (0.90–1.46)
Surgery (optimal/suboptimal)	**0.000**	1.66 (1.40–1.98)	0.328	1.10 (0.91–1.35)
LNM (yes/no)	**0.029**	1.24 (1.02–1.51)	0.283	1.12 (0.91–1.37)
Peritoneal involvement (yes/no)	**0.000**	5.35 (3.84–7.47)	**0.000**	4.14 (2.93–5.85)
Platinum sensitivity (sensitive/resistant)	**0.000**	3.92 (3.21–4.79)	**0.000**	3.23 (2.63–3.96)

Bold text indicates statistical significance at *p* < 0.05 level. Abbreviations: LNM—lymph node metastasis; FIGO—International Federation of Gynecology and Obstetrics.

**Table 6:** Conditional survival rates based on previous time survived (CS-OS) or on time without progression (CS-PFS) in patients with advanced epithelial ovarian cancer.

Time Already Survived/Progression-Free	CS-OS at 1 Year (95% CI)	CS-OS at 5 Years (95% CI)	CS-PFS at 1 Year (95% CI)	CS-PFS at 5 Years (95% CI)
6 months	87 (84–90)	49 (45–53)	89 (86–92)	44 (40–48)
1 year	88 (85–91)	49 (45–53)	90 (87–93)	46 (42–50)
3 years	91 (88–94)	57 (52–62)	93 (89–96)	54 (50–58)
5 years	95 (92–98)	66 (61–71)	95 (91–98)	62 (57–67)

Abbreviations: CS-OS = conditional survival based on overall survival; CS-PFS = conditional survival based on progression-free survival.

## Data Availability

The data presented in this study are available on reasonable request from the corresponding author. The data are not publicly available due to institutional privacy regulations and patient confidentiality.

## Supplementary Materials

The following supporting information can be downloaded at https://www.mdpi.com/article/10.3390/curroncol33010017/s1, Table S1: Applied chemotherapy protocols.
