# Supplementary material for: Shifting Survival Horizons in Advanced Ovarian Cancer: A Conditional Survival Perspective
Source: Curr Oncol. 2025 Dec 29;33(1):17. doi: 10.3390/curroncol33010017 (PMC12840376; doi:10.3390/curroncol33010017)
Supplement: Supplementary file 1 [file curroncol-33-00017-s001.zip › curroncol-3988525-supplementary.pdf]

Table S1. Applied chemotherapy protocols.

		N	%
First line		801	99,1
	Paclitaxel+Carboplatin	758	
	DUO –O trial	17	
	Paclitaxel+Carboplatin+Bevacizumab	22	
	Gemcitabine + Carboplatin	3	
	Paclitaxel+Carboplatin+Trastuzumab	1	
Second line		572	70,8
	Paclitaxel+Carboplatin	86	
	PLD + bevacizumab	88	
	Paclitaxel+Carboplatin+Bevacizumab	165	
	Gemcitabine+ Bevacizumab	6	
	PLD+Carboplatin+Bevacizumab	44	
	Gemcitabine+Platin+Bevacizumab	65	
	Letrozol	6	
	PLD+Carboplatin	45	
	PARP inhibitors	11	
	PLD	32	
	Gemcitabine	16	
	Topotecan	2	
	Gemcitabine +Carboplatin	5	
	Etoposid	1	
Third line		332	41,1
	Paclitaxel+Carboplatin	39	
	Paclitaxel + Bevacizumab	6	
	Gemcitabine + Bevacizumab	10	
	Paclitaxel+Carboplatin+Bevacizumab	16	
	PLD+Carboplatin+Bevacizumab	7	
	PLD+Bevacizumab	12	
	Gemcitabine+Platin+Bevacizumab	28	
	PLD+Carboplatin	30	
	Paclitaxel	17	
	PLD	51	
	Gemcitabine	89	
	Topotecan	17	
	PARP inhibitors	8	
	Trametinib	2	
Fourth line		203	25,1
	Topotecan	54	
	Gemcitabine	51	
	Paclitaxel	27	
	Paclitaxel + Carboplatin	22	
	PLD	18	
	PLD +Carboplatin	12	
	Paclitaxel + Carboplatin +Bevacizumab	5	
	Gemcitabine + Cysplatin	5	
	Topotekan + Bevacizumab	4	
	Docetaxel	3	
	Gemcitabine + Bevacizumab	2	
Fifth line		118	14,6
	Topotecan	36	
	Paclitaxel	24	
	Gemcitabine	17	
	Paclitaxel + Carboplatin	16	
	PLD+ Bevacizumab	6	
	PLD	5	
	Gemcitabine + Carboplatin	4	
	Paclitaxel + Bevacizumab	4	
	Megestrol asetat	3	
	Tamoxifen	2	
	Pemetrexet	1	

Sixth line		59	7,3
	Topotecan	12	
	Paclitaxel	12	
	Paclitaxel+Carboplatin	12	
	Vinorelbin	6	
	Gemcitabine	5	
	Megestrol asetat	5	
	PLD	3	
	Etoposide	2	
	PLD + Carboplatin	2	
Seventh line		21	2,6
	Topotecan	5	
	Vinorelbin	4	
	Pemetrexet	3	
	Paclitaxel	3	
	Gemcitabine	2	
	Megestrol asetat	2	
	Etoposide	1	
	Cisplatin	1	
Eighth line		9	1,1
	Vinorelbin	3	
	Pemetrexet	2	
	Topotekan	1	
	Megesterol asetat	1	
	Letrosole	1	
	Etposide	1	

Abbreviations: PLD, pegylated liposomal doxorubicin
